# Emotion Regulation in Adolescents: Evidence of the Validity and Factor Structure of the Cognitive Emotion Regulation Questionnaire (CERQ)

**DOI:** 10.3390/ijerph19063602

**Published:** 2022-03-18

**Authors:** Elena Betegón, Jairo Rodríguez-Medina, Macarena del-Valle, María Jesús Irurtia

**Affiliations:** 1 Department of Psychology, Faculty of Education and Social Work, University of Valladolid (UVa), Recognized Research Group (GIR) Psychology and Health, 47011 Valladolid, Spain; mjirurtia@uva.es; 2 Department of Pedagogy, Faculty of Education and Social Work, University of Valladolid (UVa), Recognized Research Group (GIR) Psychology and Health, 47011 Valladolid, Spain; jairo.rodriguez.medina@uva.es; 3 Institute of Basic and Applied Psychology and Technology (IPSIBAT), National Scientific and Technical Research Council (CONICET), National University of Mar del Plata (UNMDP), Recognized Research Group (GIR) Psychology and Health, Mar del Plata 7600, Argentina; mdelvalle1989@gmail.com

**Keywords:** adolescents, Cognitive Emotion Regulation Questionnaire, confirmatory factor analysis, psychometric properties, Spanish validation

## Abstract

The Cognitive Emotion Regulation Questionnaire (CERQ) is an assessment tool to evaluate cognitive emotion regulation strategies. The main objective of this study is to provide new empirical evidence about the validity and reliability of the CERQ via a sample of 271 Spanish adolescents (136 female, 135 male) aged from 15 to 18 years (M = 15.7, SD = 0.76). The analytical process was carried out in two phases. A confirmatory factor analysis was performed on the polychoric correlation matrix between items. Four possible alternative models were contrasted: two models with nine factors and two models with two second-order factors and nine first-order factors, with 36 and 27 items, respectively. The model with nine correlated factors and 27 items obtained the best indices of overall fit. Subsequently, the reliability of the measurements was estimated on this model. The results reaffirm the validity of the 27-item version of the CERQ over the original 36-item structure. The findings also confirm that the CERQ is a reliable instrument for the evaluation of emotion regulation strategies in adolescents.

## 1. Introduction

Adolescence is considered as a transition process from childhood to adulthood. It is a stage of life in which major changes take place over a short period of time [1]. This process consists of three interrelated levels [2]: biological (puberty), psychological, and social. The changes are a consequence of the developmental mechanisms and cultural demands of a specific social context, which includes defining dimensions such as social values. Due to the need to quickly adapt to these changes, various psychopathological symptoms often occur that directly affect the well-being and functioning of adolescents [3,4,5]. Numerous studies have shown that 32% of the child and adolescent population suffer from some kind of anxiety disorder generated by the presence of a stressor, such as lack of self-confidence, low self-esteem, or frustration due to poor academic performance [6,7,8,9,10]. These studies suggest that between 30% and 50% of these cases are associated to another type of disorder, such as aggressive behaviour, eating disorders, obsessive compulsive disorder, etc. [11,12,13,14]. In addition, 80% of adolescents suffering from these conditions do not receive any treatment or professional care [15].

In many cases, these symptoms are linked to the inability of individuals to recognise their emotions or to inadequate emotion regulation [16]. Emotions are an essential aspect of daily life and can promote or impair a person’s health and play a significant role in different cognitive processes. Therefore, the capacity to regulate or modulate emotional states is key to maintaining good mental health [17]. Emotion regulation is defined as the use of mechanisms, skills, and strategies with the goal of maintaining, increasing, or suppressing an existing emotional state [18]. Emotion regulation implies being aware of the state of one’s emotions and using strategies to manage mood [19,20]. Different studies have demonstrated that when people are unable to effectively regulate their emotions, distress may persist over time and may lead to different psychopathological symptoms and health problems in general [21,22,23,24]. Furthermore, gender has also been considered in many studies; females tend to present higher anxiety-related symptoms and a wider variety of mental disorders than males [6,21,25,26,27,28,29].

The growing scientific interest in the personal and interpersonal processes and skills that facilitate emotion regulation has been highlighted by the increase in publications on these topics over the last 30 years [30,31,32]. A key factor for research in this area is the availability of instruments that allow accurate assessment of the emotion regulation mechanisms.

One of the most commonly used models for understanding emotion regulation processes is that of Garnefski et al. [19], which states that emotions evoked by a negative event can be regulated by cognitive processing. Thus, the model postulates that people can use nine emotion regulation strategies: *Blaming others* (blaming another person for the occurrence of negative incidents or events), *Self-blame* (blaming oneself for the experience), *Rumination* (systematically thinking about the feelings and thoughts associated with the negative event), *Catastrophizing* (explicitly emphasising the terror of what you have experienced), *Putting into perspective* (pushing aside the severity of the event in comparison to other events), *Positive reappraisal* (creating a positive appraisal of the event in terms of personal growth), *Refocus on planning* (thinking about what steps to take and how to handle the negative event), *Positive refocusing* (thinking about joyful and pleasant issues instead of thinking about the actual event), and *Acceptance* (accepting what you experienced and resigning yourself to what has happened).

In addition, these strategies have frequently been classified into two dimensions: *adaptive cognitive strategies* of emotion regulation (Putting into Perspective, Acceptance, Positive Refocusing, Positive Reappraisal, and Refocus on Planning) and *maladaptive cognitive strategies* of emotion regulation (Rumination, Catastrophizing, Self-blame, and Blaming others).

To evaluate these nine strategies, Garnefski et al. [19] proposed the Cognitive Emotion Regulation Questionnaire (CERQ), which is the first and only emotion regulation assessment instrument that separates cognitive regulation strategies from behavioural strategies [33]. It is one of the most widely used instruments for the assessment of emotion regulation and has shown its usefulness in different fields, such as clinical [34] or educational [35,36]. For example, Giménez et al. [34] indicate that poor use of emotion regulation strategies is associated with greater perceived distress and the possibility of developing psychopathological symptoms. In this sense, the CERQ is a suitable instrument to study cognitive emotion regulation strategies and to understand their influence on subjective well-being and mental health.

The CERQ is a self-report instrument consisting of 36 items distributed according to the nine strategies of the model (four items for each dimension). Garnefski et al. [19] reported that the nine factors together explained 64.6% of the variance with communalities ranging between 0.46 and 0.73. Subsequent studies confirmed the nine-dimensional structure [37,38] and verified the validity and reliability of the scale in different populations [39,40]. The instrument was also adapted in different countries such as France [41], China [42], and Spain [43], where the nine-dimensional model showed a good fit to the data. The internal consistency indices (Cronbach’s alpha) found in these and other investigations [44] typically range between 0.60 and 0.90 points.

However, some studies show discrepancies with these results. For example, McKinnon et al. [45] reported that a five-factor model provided the best fit in an adult clinical population. In relation to the Spanish population, Carvajal et al. [33] found that reducing the scale to 27 items improved model fit. Holgado-Tello et al. [46] supported this 27-item version of the CERQ. Also in the Spanish population, Domínguez-Sánchez et al. [43] reported that an alternative model integrating the nine dimensions into two second-order factors (adaptive and maladaptive strategies) showed appropriate global fit indices.

The CERQ has also been adapted for children [37], and this adaptation has received a considerable interest in various studies, [5,47]. However, even though Garnefski et al. [19] indicated that the CERQ is designed as a self-report questionnaire that can be administered from 12 years of age, its properties have not yet been sufficiently explored in the Spanish-speaking adolescent population. Given the importance of cognitive emotion regulation, the CERQ could constitute a conceptually sound instrument for assessing underlying cognitive coping processes in adolescence. Therefore, the main objective of this study is to provide new empirical evidence on the validity and reliability of the CERQ in a sample of Spanish adolescents.

## 2. Materials and Methods

### 2.1. Participants

A total of 271 Spanish compulsory secondary school students (Educación Secundaria Obligatoria, ESO) participated in this study (136 females 50.18%, 135 males 49.82%). Ages ranged from 15 to 18 years (M = 15.7, SD = 0.76). The participants attended two different secondary schools in the city of Valladolid, Spain. Non-probability convenience sampling was employed as, after contacting different schools in urban areas, a working relationship was established with the schools with the highest level of educational innovation.

### 2.2. Instruments

The Spanish adaptation of the Cognitive Emotion Regulation Questionnaire (CERQ-S; [43] was employed. This tool consists of 36 items distributed into nine subscales related to emotion regulation strategies. This questionnaire has shown to be reliable and valid in prior studies with both Spanish [32,43] and international populations [37,44,48,49,50].

The 36 items refer to the nine cognitive strategies of emotion regulation: Rumination, Catastrophizing, Self-blame, Blaming others, Putting into Perspective, Acceptance, Positive Refocusing, Positive Reappraisal, and Refocus on Planning [37]. Each of these subscales is made up of four items in the 36-item tool and three items in the 27-item tool.

High scores in each of the subscales indicate greater use of that strategy. Scores are obtained from participants’ responses about their use of each strategy on a Likert scale (1 = almost never; 5 = almost always). The internal consistency of each subscale oscillated between alphas of 0.68 and 0.89 in the original validation [19]. Scores obtained in the Spanish adaptation [43] were similar (between 0.61 and 0.89).

### 2.3. Procedure

In all the procedures, the ethical standards of the institutions, the criteria of the National Research Committee [Comité Nacional de Investigación], and the international criteria of the APA [51] and the 1964 Declaration of Helsinki [52] (as well as their subsequent amendments or similar ethical rules), were observed. Within these ethical principles for research involving human subjects, the confidentiality of the data and the pursuit of the benefit of the participants are ensured. Furthermore, as this was a study with adolescents, their parents or legal guardians gave prior informed consent for their children to participate and to be assessed.

The evaluation protocol was administered in group sessions with 20 to 30 students who were divided into their academic year groups within their classrooms. An examiner was always present to clarify any doubt that might arise.

### 2.4. Data Analysis

The analytical process was carried out in two phases. In phase 1, a confirmatory factor analysis (CFA) was performed on the polychoric correlation matrix between items [53] after verifying the fit of the data for the factor analysis using the Kaiser–Meyer–Olkin test (KMO) and Bartlett’s sphericity test (KMO = 0.75; Bartlett’s sphericity test, χ² (630) = 1676.94; *p* < 0.001). Following some suggestions in previous literature concerning the Spanish population [33,43,46], four models were estimated: two models with nine correlated factors with 36 and 27 items, (models 1 and 2, respectively), and two models with two second-order factors (models 3 and 4, respectively) combining five first-order factors (related to *adaptative strategies* of emotion regulation) and four first-order factors (related to *maladaptive strategies* of emotion regulation), with 36 and 27 items, respectively.

In the second phase, the reliability of the measures (internal consistency, reliability of the individual indicators, construct reliability, and observational error) was estimated using the model with nine correlated factors and 27 items (model 3). All models were estimated via diagonally least weighted squares on the polychoric correlation matrix using R 3.6.3 software [54] and the lavaan package [55]. All models were estimated using weighted least squares (WLSMV).

The goodness of fit was contrasted using the comparative fit index (CFI), the Tucker-Lewis index (TLI), and the root mean square error of approximation (RMSEA). It is considered that CFI and TLI indices greater than 0.90 indicate acceptable fit, while a value higher than 0.95 is considered good [56]. In the case of the RMSEA, values of 0.05 or less are considered good and less than 0.08 is considered acceptable [56,57]. Moreover, following the recommendations of Chen [58] and Cheung and Rensvold [59], increases of less than 0.010 in CFI and TLI and decreases of less than 0.015 in RMSEA suggest no relevant changes in the fit of one model compared with the following more restrictive model.

## 3. Results

**Figure 1:**
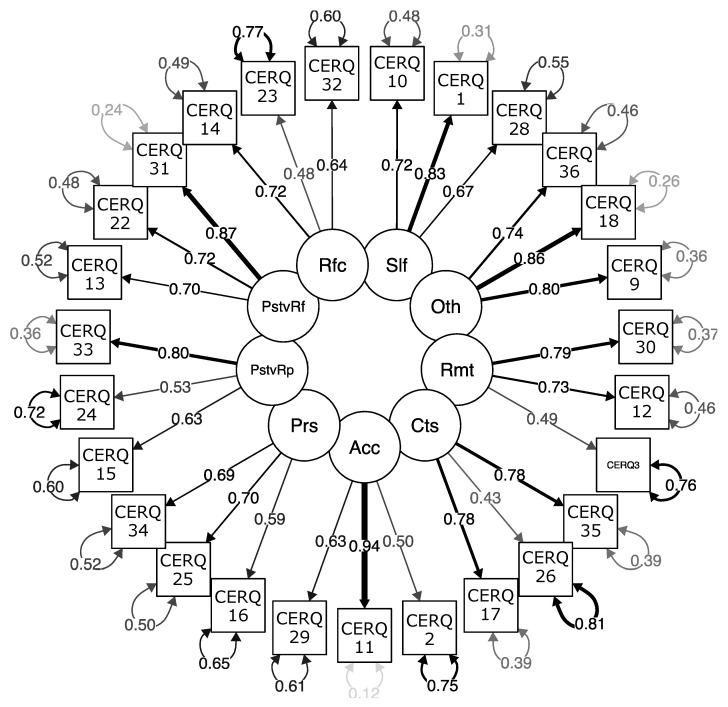
Measurement model with nine correlated factors and 27 items. Note: complete standardised model for the 27-item Spanish version of the Cognitive Emotion Regulation Questionnaire (CERQ-S-27; Model 3). Slf = Self-blame; Acc = Acceptance; Rmt = Rumination; PstvRf = Positive Refocusing; Rfc = Refocus on planning; PstvRp = Positive Reappraisal; Prs = Putting into perspective; Cts = Catastrophizing; Oth = Blaming others.

The results of the CFA allowed comparison of the goodness of fit of four alternative models: two with nine factors and two with two second-order factors and nine first-order factors, with 36 and 27 items respectively. The result was extremely favourable for model 3 with nine correlated factors and 27 items. As can be observed in Table 1, the improvement in the fit of model 3 over the other models proved conclusive. Figure 1 shows the graphical representation of the measurement model.

Global alpha ordinal reliability values [60] of α = 0.83 and McDonald’s omega values [61,62] of ω = 0.82 were obtained. Both values are adequate. Good internal consistency indices were obtained for *adaptive strategies* (α1 = 0.87; ω1 = 0.9) and for *maladaptive strategies* (α2 = 0.82; ω2 = 0.89).

The composite reliability (CR) analysis of each latent variable provides an indicator of the reliability of the construct [63]. In all cases, CR was higher than 0.70 (CR1 = 0.78; CR2 = 0.84; CR3 = 0.79; CR4 = 0.71; CR5 = 0.74; CR6 = 0.71; CR7 = 0.79; CR8 = 0.81; and CR9 = 0.74). Therefore, it can be concluded that the indicators of the nine factors are a reliable measurement of the construct. Average variance extracted (AVE) showed values higher than or extremely close to 0.5 in all cases (AVE1 = 0.55; AVE2 = 0.64, AVE3 = 0.47, AVE4 = 0.47, AVE5 = 0.51; AVE6 = 0.44, AVE7 = 0.44, AVE8 = 0.58, and AVE9 = 0.48). Thus, it can be concluded that a substantial amount of the variance of the indicators is explained by the construct compared with the error of the measurement. All these indicators constitute evidence of reliability in the operationalisation of the nine latent variables of the scale.

The reliability of each indicator can be checked using the R2 values, which indicate the proportion of variance of each indicator that explains the latent variable (high R2 values indicate that the indicator is reliable). The most reliable indicator for the *acceptance* factor was item 11 (*I think that I have to accept the situation*; R2 = 0.88). The most reliable indicator for the *positive refocusing* factor was item 31 (*I think of pleasant things that have nothing to do with it*; R2 = 0.757). The most reliable indicator for the *positive reappraisal* factor was item 31 *I look for the positive sides to the matter* (R2 = 0.639). The item 29 *I feel that others are responsible for what has happened* proved to be the most reliable indicator for the *blaming others* factor (R2 = 0.735). The item 1 *I feel that I am the one to blame for it* proved to be the most reliable indicator for the *self-blame* factor (R2 = 0.694).

Regarding convergent validity (e.g., the indicators of each latent variable have a high shared variance), Table 2 and Table 3 indicate that (a) the factor loadings of all of the indicators proved significant; (b) all but three indicators (*I often think about how I feel about what I have experienced*—λ = 0.493; *I often think that what I have experienced is the worst that can happen to a person*—λ = 0.435; *I think about how to change the situation*—λ = 0.478) higher than 0.5; and (c) the AVE of the saturations of the items in each factor are all those which are higher or extremely close to 0.5.

The evidence of discriminant validity demonstrates that each of the constructs analysed is unique and different from other constructs. To test discriminant validity, four approaches were used [53]. First, the correlation between each pair of factors was set to 1, and the fit of the resulting models was compared to the fit of the original model of nine correlated factors and 27 items. The results showed that this model was significantly superior to the models in which the correlation between each pair of factors was set to 1 (Table A1). Second, the confidence interval test [64] demonstrated that the confidence interval of the correlations between the factors does not contain 1 (Table 4). Third, it was verified that the HTMT ratio [65] of the correlations between the indicators of different factors (heterotrait-heteromethod correlations–HT) and between the correlations of the indicators of the same factor (monotrait-heteromethod correlations–MT) is less than 0.9 (F1–F2, HT/MT = 0.771; F1–F3, HT/MT = 0.693; F2–F3, HT/MT = 0.835).

Fourth, Fornell and Larcker’s criterion [63] states that AVE from each factor higher than the square of the correlations between each pair of factors can be considered as evidence of discriminant validity. This criterion is fulfilled in all but two cases (Table 5). Specifically, AVE of factors 6 (Perspective, AVE = 0.444) and 7 (Reappraisal, AVE = 0.440) was lower than the coefficient of determination between them both (ρ2 = 0.513). Likewise, AVE of factors 7 (Reappraisal, AVE = 0.440) and 9 (Planning, AVE = 0.482) was lower than the coefficient of determination between them both (ρ2 = 0.673).

## 4. Discussion

The main objective of this study is to provide new empirical evidence on the validity and reliability of the CERQ [19,31] in a sample of Spanish adolescents. The CERQ is an emotion regulation assessment tool that allows discrimination between cognitive and behavioural strategies [33]. Prior studies have shown empirical evidence of the psychometric properties of the CERQ in the Spanish population [32,33,43,46,66,67]. However, less attention has been paid to its properties in the adolescent population. For this reason, the psychometric properties of the CERQ were analysed in a sample of students from the city of Valladolid (Spain).

According to some proposals in the literature, four models were estimated through CFA. The results obtained suggest the reduction of the scale to 27 items (model 3) distributed in nine factors (strategies). The nine-dimensional structure coincides with that originally proposed by Garnefski et al. [19]. However, the number of items suggested in the results differs from the original instrument and rather supports the proposal of Carvajal et al. [33]. In this sense, these authors recommend adapting the Spanish version to a shorter version called CERQ-S-27 and another even shorter version of 18 factors (ERQ-short), which has already been validated in other countries [38,48,50,68,69,70,71,72]. Given that the sample used in the present study is also Spanish, it is reasonable that the results regarding the number of items are more similar to previous studies carried out in this country.

Regarding the grouping into functional/adaptive and dysfunctional/maladaptive strategies, the results suggest the importance of maintaining the multidimensionality of the CERQ. Thus, it is recommended not to merge the scores of the different strategies as reported in other studies [36,39,49]. However, the fit of model 4 (27 items with two second-order factors and nine first-order factors) has also demonstrated appropriate global fit indices which should be considered in future research. Although it is better to address the scales separately, using them as a whole may have interesting implications in certain specific contexts, as already pointed out by Domínguez-Sánchez et al. [43] and D’Augerot et al. [36].

The results suggest that the nine subscales of the CERQ present adequate internal consistency, sufficient reliability and adequate construct reliability. Furthermore, sufficient evidence of convergent validity and discriminant validity was obtained. However, discriminant validity points to the possible convenience of merging some factors (Perspective-Appraisal-Planning), but this would require a much larger sample and a theory to support this hypothesis.

In short, these results confirm the need for further research on the dimensionality of the CERQ and cognitive emotion regulation strategies. Psychometric properties were generally similar to those of the original Spanish adaptation, confirming the validity of the short 27-item version. This conclusion has been reached not only in studies with national sampling, but also in other international research, such as the Italian [73] or German [74] adaptations.

Finally, the results of the present study should be interpreted in light of certain limitations. First, the sample was non-probabilistic, limiting the generalizability of the results. It would be of great interest to employ a random sampling method to be able to generalise the results. Second, the exclusive use of self-report methods also imply the possibility of assessment bias [75]. Third, because there are few studies on the CERQ functioning in adolescent populations, comparison of the findings was limited. Finally, although the number of participants was sufficient to perform the CFA and achieve the objective of the study, a larger sample would improve the accuracy of the results and give them greater scope. Moreover, a larger number of participants would enable additional analyses such as a gender factorial invariance analysis. As females tend to present a higher prevalence of anxiety-related symptoms and a wider variety of mental disorders than males [6,21,25,26,27,28,29,34,35], examining validity and reliability of the CERQ across gender would be of great interest. It is expected that future studies may address this limitation.

## 5. Conclusions

Despite these issues, the present study represents a significant contribution, since the findings support the use of the CERQ in the adolescent population, as well as its usefulness, validity, and reliability. Due to the implications of cognitive emotion regulation mechanisms on the quality of life of individuals, future studies should further explore this and other instruments that assess these processes. In this way, professionals would have appropriate instruments to assess adolescents’ coping strategies and, consequently, propose interventions to help them manage stressful situations. It would also be possible to investigate the relationship and influence of these strategies on psychopathological symptoms in order to improve the overall health of adolescents [76].

The ability to regulate one’s emotions not only affects the emotions a person experiences at a specific time, but also how they feel and express them [18]. Emotion regulation plays a fundamental role in both cognitive and psychological functioning within the field of mental health. However, despite the wide range of research on emotion regulation at different stages of life, only a small proportion of this research focuses on adolescence [77]. As previously mentioned, adolescence is characterised by physical, psychological, and social changes, which can lead to emotion dysregulation. All these changes affect not only emotions and psychopathology, but also other aspects, such as academics [49]. Therefore, it is of great importance to further investigate this ability at this developmental stage.

## Figures and Tables

**Table 1:** Comparison of the fit indices of the four models considered.

Model	χ² (df)	*p*(χ²)	RMSEA	RMSEA 90% CI	SRMR	CFI	TLI
1	1024.68 (558)	<0.001	0.066	[0.050–0.071]	0.083	0.912	0.901
2	1487.14 (584)	<0.001	0.076	[0.071–0.080]	0.099	0.830	0.817
3	356.74 (288)	=0.004	0.030	[0.018–0.039]	0.064	0.981	0.977
4	597.68 (314)	<0.001	0.058	[0.051–0.065]	0.083	0.922	0.912

Note. Model 1 = 36 items and 9 correlated factors; Model 2 = 36 items, 2 s-order factors and 9 first-order factors; Model 3 = 27 items and 9 correlated factors; Model 4 = 27 items, 2 s-order factors and 9 first-order factors. RMSEA = root mean square error of approximation; CFI = comparative fit index; TLI = Tucker–Lewis index; df = degrees of freedom; CI = confidence interval.

**Table 2:** Estimates of the nine-factor solution (maladaptive strategies).

Latent Factor	Indicator	B	SE	Z	*p*-Value	Beta
Self-blame	I feel that I am the one to blame for it	0.831	0.058	14.275	0	0.833
Self-blame	I feel that I am the one who is responsible for what has happened	0.747	0.056	13.313	0	0.722
Self-blame	I think that basically the cause must lie within myself	0.640	0.050	12.808	0	0.670
Blaming others	I feel that others are to blame for it	0.759	0.059	12.789	0	0.802
Blaming others	I feel that others are responsible for what has happened	0.872	0.068	12.919	0	0.858
Blaming others	I feel that basically the cause lies with others	0.739	0.060	12.405	0	0.738
Rumination	I often think about how I feel about what I have experienced	0.583	0.052	11.194	0	0.493
Rumination	I am preoccupied with what I think and feel about what I have experienced	0.776	0.054	14.273	0	0.734
Rumination	I dwell upon the feelings the situation has evoked in me	0.947	0.064	14.745	0	0.793
Catastrophising	I keep thinking about how terrible it is what I have experienced	0.931	0.057	16.198	0	0.778
Catastrophising	I often think that what I have experienced is the worst that can happen to a person	0.468	0.042	11.077	0	0.435
Catastrophising	I continually think how horrible the situation has been	0.957	0.059	16.231	0	0.783

Note. B = Estimate Factor Loadings; SE = Standard Error; Beta = Standardized Factor Loadings.

**Table 3:** Estimates of the nine-factor solution (adaptive strategies).

Latent Factor	Indicator	B	SE	Z	*p*-Value	Beta
Acceptance	I think that I have to accept that this has happened	0.532	0.050	10.647	0	0.501
Acceptance	I think that I have to accept the situation	0.899	0.064	14.145	0	0.940
Acceptance	I think that I must learn to live with it	0.669	0.052	12.805	0	0.626
Perspective	I think that other people go through much worse experiences	0.723	0.053	13.730	0	0.594
Perspective	I think that it hasn’t been too bad compared to other things	0.747	0.050	14.849	0	0.704
Perspective	I tell myself that there are worse things in life	0.802	0.055	14.680	0	0.695
Positive reappraisal	I think that I can become a stronger person as a result of what has happened	0.721	0.047	15.403	0	0.630
Positive reappraisal	I think that the situation also has its positive sides	0.617	0.045	13.829	0	0.532
Positive reappraisal	I look for the positive sides to the matter	0.906	0.054	16.643	0	0.799
Positive refocusing	I think of pleasant things that have nothing to do with it	0.852	0.051	16.585	0	0.696
Positive refocusing	I think of pleasant things that have nothing to do with it	0.818	0.048	16.989	0	0.723
Positive refocusing	I think of pleasant things that have nothing to do with it	0.997	0.055	18.180	0	0.870
Planning	I think about how I can best cope with the situation	0.684	0.050	13.586	0	0.717
Planning	I think about how to change the situation	0.445	0.041	10.960	0	0.478
Planning	I think of a plan of what I can do best	0.686	0.052	13.124	0	0.635

Note. B = Estimate Factor Loadings; SE = Standard Error; Beta = Standardized Factor Loadings.

**Table 4:** Correlations between factors (solution of nine correlated factors and 27 items).

	F2 (SE)	F3 (SE)	F4 (SE)	F5 (SE)	F6 (SE)	F7 (SE)	F8 (SE)	F9 (SE)
F1	0.025 (0.038)	0.502 (0.051)	0.530 (0.052)	−0.068 (0.045)	0.022 (0.045)	−0.163 (0.047)	−0.191 (0.04)	−0.061 (0.047)
F2		0.109 (0.038)	0.401 (0.044)	−0.031 (0.039)	0.194 (0.043)	0.077 (0.04)	0.206 (0.037)	0.084 (0.045)
F3			0.662 (0.059)	0.092 (0.047)	0.207 (0.05)	−0.027 (0.049)	0.047 (0.042)	0.141 (0.053)
F4				−0.234 (0.047)	−0.072 (0.048)	−0.343 (0.051)	−0.165 (0.043)	−0.214 (0.053)
F5					0.443 (0.055)	0.432 (0.056)	0.330 (0.044)	0.465 (0.059)
F6						0.716 (0.068)	0.485 (0.051)	0.637 (0.072)
F7							0.627 (0.054)	0.820 (0.081)
F8								0.478 (0.057)

Note. F1 = Self-blame; F2 = Blaming others; F3 = Rumination; F4 = Catastrophizing; F5 = Acceptance; F6 = Perspective; F7 = Positive reappraisal; F8 = Positive refocusing; F9 = Planning.

**Table 5:** Criterion of Fornell & Larcker [63].

	F1	F2	F3	F4	F5	F6	F7	F8	F9
Self-blame	*0.554*								
Blaming others	0.001	*0.641*							
Rumination	0.252	0.012	*0.470*						
Catastrophizing	0.28	0.16	0.438	*0.469*					
Acceptance	0.005	0.001	0.009	0.055	*0.509*				
Perspective	0	0.038	0.043	0.005	0.196	*0.444*			
Reappraisal	0.027	0.006	0.001	0.117	0.187	0.513	*0.440*		
Refocusing	0.036	0.042	0.002	0.027	0.109	0.235	0.393	*0.588*	
Planning	0.004	0.007	0.02	0.046	0.216	0.406	0.673	0.229	*0.482*

Note. F2 = Blaming others; F3 = Rumination; F4 = Catastrophizing; F5 = Acceptance; F6 = Perspective; F7 = Positive reappraisal; F8 = Positive refocusing; F9 = Planning; AVE in diagonal (in italics), coefficient of determination (ρ2) below.

## Data Availability

The data presented in this study are available on request from the corresponding author. The data are not publicly available due to privacy reasons.

## Appendix A

**Table A1:** Comparison of the fit indices of the 37 nine-factor models.

Model	χ² (df)	Δχ² (Δdf)	p(Δχ²)	RMSEA	ΔRMSEA	CFI	ΔCFI	TLI	ΔTLI
Model 3	356.74 (288)	-	-	0.030	-	0.981	-	0.977	-
1 (F1–F2)	520.80 (289)	164.06 (1)	<0.001	0.055	0.025	0.936	−0.045	0.922	−0.055
2 (F1–F3)	406.09 (289)	49.35 (1)	<0.001	0.039	0.009	0.968	−0.013	0.961	−0.016
3 (F1–F4)	400.19 (289)	43.45 (1)	<0.001	0.038	0.008	0.969	−0.012	0.963	−0.014
4 (F1–F5)	-	-	-	-	-	-	-	-	-
5 (F1–F6)	482.68 (289)	125.93 (1)	<0.001	0.050	0.02	0.946	−0.035	0.935	−0.042
6 (F1–F7)	-	-	-	-	-	-	-	-	-
7 (F1–F8)	-	-	-	-	-	-	-	-	-
8 (F1–F9)	-	-	-	-	-	-	-	-	-
9 (F2–F3)	499.59 (289)	145.85 (1)	<0.001	0.052	0.022	0.942	−0.039	0.929	−0.048
10 (F2–F4)	434.12 (289)	77.37 (1)	<0.001	0.043	0.013	0.960	−0.021	0.951	−0.026
11 (F2–F5)	-	-	-	-	-	-	-	-	-
12 (F2–F6)	466.89 (289)	110.14 (1)	<0.001	0.048	0.018	0.951	−0.03	0.940	−0.037
13 (F2–F7)	480.45 (289)	123.71 (1)	<0.001	0.050	0.02	0.947	−0.034	0.936	−0.041
14 (F2–F8)	535.64 (289)	178.9 (1)	<0.001	0.056	0.026	0.932	−0.049	0.917	−0.06
15 (F2–F9)	436.69 (289)	79.95 (1)	<0.001	0.044	0.014	0.959	−0.022	0.950	−0.027
16 (F3–F4)	377.96 (289)	21.22 (1)	<0.001	0.034	0.004	0.975	−0.006	0.970	−0.007
17 (F3–F5)	485.04 (289)	128.3 (1)	<0.001	0.050	0.02	0.946	−0.035	0.934	−0.043
18 (F3–F6)	457.76 (289)	101.02 (1)	<0.001	0.047	0.017	0.953	−0.028	0.943	−0.034
19 (F3–F7)	-	-	-	-	-	-	-	-	-
20 (F3–F8)	504.25 (289)	147.51 (1)	<0.001	0.053	0.023	0.941	−0.04	0.928	−0.049
21 (F3–F9)	432.94 (289)	76.19 (1)	<0.001	0.043	0.013	0.960	−0.021	0.952	−0.025
22 (F4–F5)	-	-	-	-	-	-	-	-	-
23 (F4–F6)	-	-	-	-	-	-	-	-	-
24 (F4–F7)	-	-	-	-	-	-	-	-	-
25 (F4–F8)	-	-	-	-	-	-	-	-	-
26 (F4–F9)	-	-	-	-	-	-	-	-	-
27 (F5–F6)	406.52 (289)	49.77 (1)	<0.001	0.039	0.009	0.968	−0.013	0.961	−0.016
28 (F5–F7)	408.05 (289)	51.30 (1)	<0.001	0.039	0.009	0.967	−0.014	0.960	−0.017
29 (F5–F8)	446.91 (289)	90.16 (1)	<0.001	0.045	0.015	0.956	−0.025	0.947	−0.023
30 (F5–F9)	394.29 (289)	37.54 (1)	<0.001	0.037	0.007	0.971	−0.010	0.965	−0.012
31 (F6–F7)	369.06 (289)	12.32 (1)	<0.001	0.032	0.002	0.978	−0.003	0.973	−0.004
32 (F6–F8)	408.90 (289)	52.15 (1)	<0.001	0.039	0.009	0.967	−0.014	0.960	−0.017
33 (F6–F9)	372.65 (289)	15.91 (1)	<0.001	0.033	0.003	0.977	−0.004	0.972	−0.005
34 (F7–F8)	385.30 (289)	28.55 (1)	<0.001	0.035	0.005	0.973	−0.008	0.968	−0.009
35 (F7–F9)	360.62 (289)	3.87 (1)	=0.049	0.030	0	0.980	−0.001	0.976	−0.001
36 (F8–F9)	394.56 (289)	37.81 (1)	<0.001	0.037	0.007	0.971	0.010	0.965	0.012

Note. RMSEA = root mean square error of approximation; CFI = comparative fit index; TLI = Tucker–Lewis index; df = degrees of freedom; CI = confidence interval; Δ (CFI, TLI, RMSEA) = changes in fit with respect to model 3; F1 = Self-blame; F2 = Blaming others; F3 = Rumination; F4 = Catastrophizing; F5 = Acceptance; F6 = Perspective; F7 = Positive reappraisal; F8 = Positive refocusing; F9 = Planning.
